# Unraveling the Trio of Interrupted Descending Aorta, Aortic Stenosis, and Bicuspid Aortic Valve: A Complex Cardiovascular Conundrum

**DOI:** 10.7759/cureus.49365

**Published:** 2023-11-24

**Authors:** Saket Toshniwal, Gajendra Agrawal, Anuj Chaturvedi, Akash Lohakare, Sunil Kumar

**Affiliations:** 1 Medicine, Jawaharlal Nehru Medical College, Datta Meghe Institute of Higher Education and Research, Wardha, IND; 2 Cardiology, Jawaharlal Nehru Medical College, Datta Meghe Institute of Higher Education and Research, Wardha, IND

**Keywords:** interrupted aortic arch, adult onset, congenital heart disease, bicuspid aortic valve, aortic stenosis, interrupted descending aorta

## Abstract

Interrupted descending aorta (IDA) is an extremely rare congenital heart defect characterized by a complete loss of connection between the ascending and descending aorta. This condition is typically diagnosed in infancy or early childhood, but there have been very few cases reported in adulthood. Here, we present a unique case of an IDA in a 16-year-old patient with concomitant aortic stenosis (AS) and bicuspid aortic valve (BAV), making it an extremely rare scenario. This case highlights the importance of early diagnosis and appropriate management in patients with an IDA, particularly when in association with other cardiovascular abnormalities.

## Introduction

The prevalence of interrupted descending aorta (IDA) is approximately 1% of all congenital heart diseases, affecting three per million live births, making it an extremely rare anomaly, and its confluence with other structural anomalies like severe aortic stenosis (AS) and bicuspid aortic valve (BAV) makes it a rare clinical scenario, making it a complex cardiovascular conundrum, as presented in our case. While most cases are diagnosed in infancy or early childhood, very few adult-onset cases have been reported [1]. In this complex scenario, multiple congenital anomalies converge to create a challenging clinical condition. Interrupted aortic arch (IAA) disrupts the natural continuity of the aorta at different levels, necessitating surgical intervention to restore normal aortic blood flow. This condition is typically classified into three types based on the specific interruption site: type A, distal to the left subclavian artery origin (IDA); type B, between the left common carotid artery and the left subclavian artery; and type C, occurrence between the innominate artery and the left common carotid artery [2,3]. Simultaneously, the presence of other cardiovascular anomalies, along with an IDA, can impose greater management challenges and potential complications.

## Case presentation

A 16-year-old female child presented with complaints of shortness of breath on mild exertion with headache and recurrent fever and cough as well as generalized weakness and failure to thrive. On general examination, an elevated blood pressure of 160/90 mm Hg was found in both the upper extremities, while lower extremity blood pressure measured 90/60 mm Hg bilaterally, without other significant findings. Her heart rate was 90 beats per minute with normal sinus rhythm on the electrocardiogram. A continuous systolic murmur was heard all over the heart.

**Figure 1:**
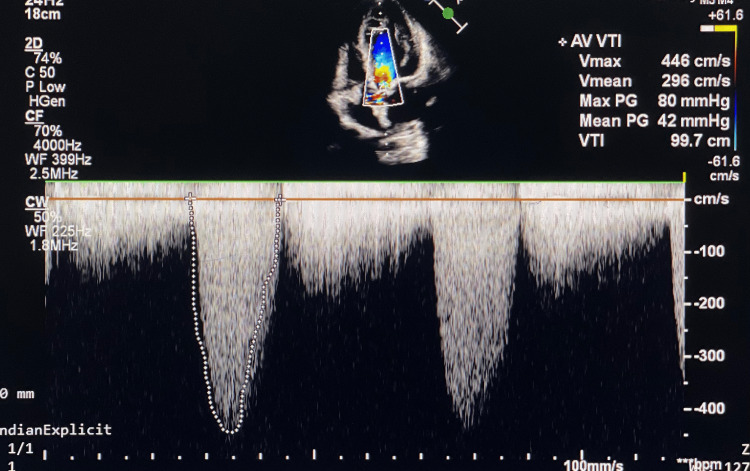
Severe aortic stenosis on 2D echocardiography 2D: two-dimensional Pulsed-wave Doppler echocardiogram on transthoracic, apical, five-chamber view showing AVmax of 446 cm/seconds with peak gradient across the aortic valve of 80 mm Hg and mean gradient of 42 mm Hg depicting severe aortic stenosis

On a two-dimensional (2D) echocardiogram, the presence of severe AS, as shown in Figure 1, was found along with a BAV and a dilated ascending root of the aorta.

**Figure 2:**
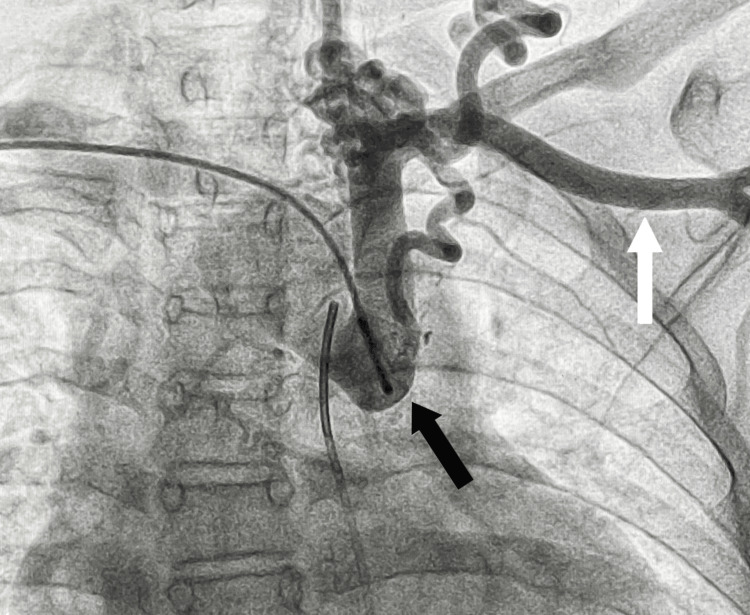
IDA IDA: interrupted descending aorta Invasive angiogram under fluoroscopy showing IDA (black arrow) proximally at the level of the left subclavian artery (white arrow) with collateral formation

**Figure 3:**
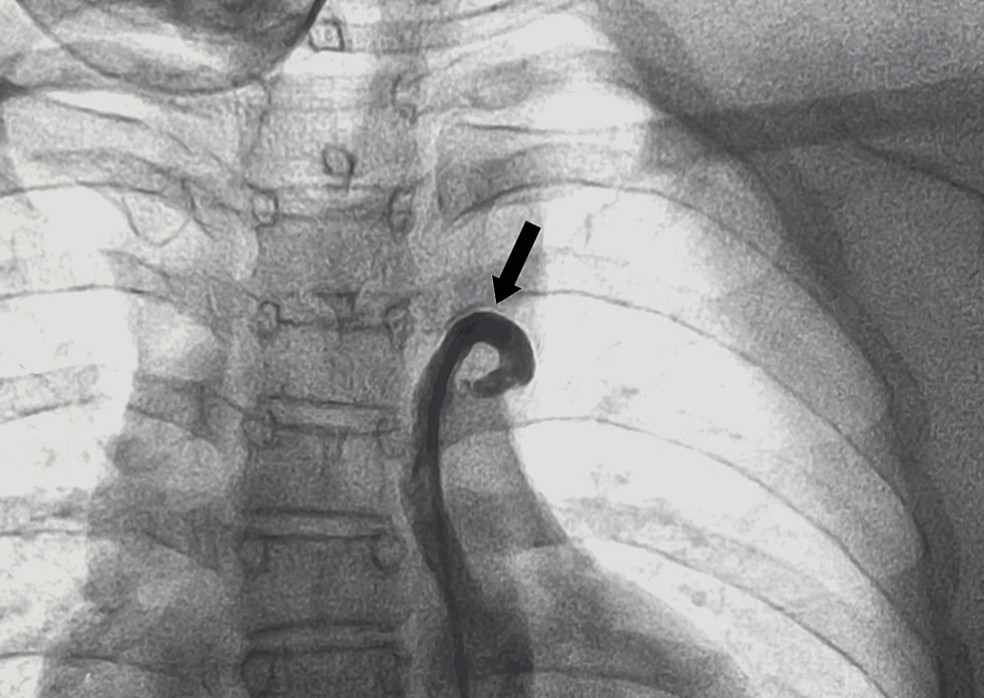
Distal end of IDA IDA: interrupted descending aorta Invasive angiogram under fluoroscopy showing IDA distally (black arrow) with no proximal connection

On further evaluation, invasive angiogram through the catheterization of the femoral artery under fluoroscopy of the patient revealed IDA type A, as shown in Figure 2 and Figure 3.

A rare and complex cardiovascular situation was diagnosed, and elective balloon aortic valvuloplasty was performed without any complications. The patient was discharged after stabilization on anti-hypertensive, single anti-platelet, and statin medications with a future referral to cardiovascular surgery for the further management of the IDA.

## Discussion

IDA is a rare congenital heart defect that presents unique diagnostic and therapeutic challenges, especially when associated with other cardiovascular abnormalities such as AS and BAV, as in our case. The disease is usually diagnosed in infancy or early childhood with very few reports in adults.

22q11.2 deletions, often known as DiGeorge syndrome, are the cause of IAA in about 50% of cases. In between 75% and 85% of cases, congenital heart disease resulting from a 22q11.2 deletion might be asymptomatic or extremely severe, necessitating early intervention during the neonatal stage. The branchial arch defect and the conotruncal defect are two more congenital cardiac conditions that these patients may have in addition to an IAA. IAA, aortic coarctation, and anomalies of the right aortic arch are examples of branchial arch defects. Tetralogy of Fallot, sub-arterial ventricular septal defects, double-outlet right ventricle, and truncus arteriosus are examples of conotruncal defects; coloboma, heart disease, atresia of the choanae, retarded growth and mental development, genital anomalies, and ear malformations and hearing loss (CHARGE) syndrome is another syndrome linked to IAA [4]. The anatomical features of IDA include a complete loss of connection between the distal transverse aorta and the descending aorta, resulting in a separation between these two portions of the aorta.

The clinical manifestations of IAA can vary according to the age group and other associated cardiac anomalies, as shown in Table 1 [5-7].

**Table 1:** Clinical manifestations of IAA in different age groups associated with other cardiac anomalies IAA: interrupted aortic arch The clinical manifestations of IAA can vary depending on the severity of the interruption, age groups, and associated cardiac abnormalities [4-6]

Case	Age group	Clinical manifestation	Associated abnormalities
1	Infant	Poor feeding, failure to thrive, respiratory distress, cyanosis	Bicuspid aortic valve
2	Infant and neonates	Cardiogenic shock, acidemia	Ventricular septal defect, patent ductus arteriosus
3	Childhood	Asymptomatic	None
4	Adolescence	Dyspnea on exertion, chest pain, refractory hypertension	Coarctation of the aorta
5	Adult	Refractory hypertension, acute paraplegia, subarachnoid hemorrhage	Aortic stenosis, ascending aortic dilatation

The diagnosis of an IDA involves a clinical evaluation involving imaging studies and diagnostic tests. A thorough physical examination can reveal important clues, such as unequal blood pressures between the upper and lower extremities or diminished or absent femoral pulses. Imaging studies such as echocardiography, magnetic resonance imaging (MRI), computed tomography angiography (CTA), and recently dual-source computed tomography (DSCT) are being widely used as evaluation studies [8]. CTA is the gold standard for evaluating aortic interruption [9]. Early diagnosis of congenital heart defects including evaluation of the aorta and coronaries is important for better management strategies [10,11]. The management of an IAA typically involves surgical interventions to restore the continuity of the aortic arch as well as addressing any associated cardiac abnormalities. Introduction of prostaglandin E1 in the management of IAA in infancy has revolutionized the treatment of IAA and improved outcomes significantly by guaranteeing perfusion of the lower half of body and avoiding acidotic insult to the infant by maintaining the patency of the ductus arteriosus and thus help in resuscitating the infant [12]. The specific surgical approach depends on the location of the interruption, the presence of additional cardiac defects, and the age and overall health of the patient. Surgical options may include aortic arch reconstruction, ventricular septal defect closure, aortic valve repair or replacement, and other procedures aimed at improving cardiovascular function [13]. The severity of the IDA, the presence of accompanying cardiac problems, the age at diagnosis, and the prompt implementation of appropriate therapeutic measures all affect the prognosis. The results for patients with IDA have improved dramatically over time, thanks to improvements in surgical methods and postoperative care. To monitor for potential problems and optimize heart function, however, continued care and long-term follow-up are crucial [14].

## Conclusions

Confluence of AS, BAV, and IDA provides a complex and difficult clinical condition. For an accurate diagnosis, prompt intervention, and long-term management of these patients, a multidisciplinary strategy combining cardiologists, cardiac surgeons, and imaging specialists is essential. To better understand the underlying causes and improve therapeutic approaches for this unusual combination of anatomical findings, additional studies are required; and hence, it is important to highlight such rare complex cases in the literature for such cases are scarce.
